# Comparison of efficacy of combination therapy of an Intravitreal injection of bevacizumab and photocoagulation versus Pan Retinal Photocoagulation alone in High risk Proliferative Diabetic Retinopathy

**DOI:** 10.12669/pjms.37.1.3141

**Published:** 2021

**Authors:** Rebecca, Fahad Feroz Shaikh, Shafi Muhammad Jatoi

**Affiliations:** 1 Dr. Rebecca, MBBS. Department of Ophthalmology, Isra University Hospital, Hyderabad, Pakistan; 2 Dr. Fahad Feroz Shaikh, FCPS, FVR, FRCS. Department of Ophthalmology, Isra University Hospital, Hyderabad, Pakistan; 3 Dr. Shafi Muhammad Jatoi, FCPS. Department of Ophthalmology, Isra University Hospital, Hyderabad, Pakistan

**Keywords:** High Risk PDR, Intravitreal bevacizumab, Panretinal photocoagulation, Regression of retinal neovessels

## Abstract

**Objective:**

To compare efficacy of intravitreal bevacizumab augmented with Panretinal photocoagulation versus Panretinal photocoagulation alone in high risk proliferative diabetic retinopathy.

**Methods:**

This is Randomized clinical control trial study conducted at ISRA University Hospital, Hyderabad from July 2018 to December 2018. A total of 76 eyes were randomized into two groups, 38 eyes undergone PRP plus intravitreal bevacizumab, while 38 eyes had PRP alone. Status of neovessels was assessed before and after treatment with the help of fundus fluorescein angiography. Neovessels at disc (NVD’s) and neovessels elsewhere (NVE’s) were assessed with the disc surface diameter.

**Results:**

Seventy-six eyes were enrolled in this randomized clinical trial into two groups consecutively, that all completed the six months follow-up. In the PRP group mean BCVA (logMAR) worsened significantly from mean 0.30±0.07 to mean 0.40±0.04 at a 30^th^ day and mean 0.40±0.04 at day 90. While BCVA become improved from 0.30±0.05 to 0.1±0.03 at week four and 0.1±0.02 at week 12 in PRP-Plus group. There was significant change in regression of NVES in PRP only group at week 4 is 2.25±0.75 (*p*=0.00004) and at 12 weeks 2.00±0.50 (*p*=0.00002), while in PRP + intravitreal bevacizumab group at 4^th^ week was 1±0.5 (*p* =0.0001) and at 12^th^ week was 0.75±0.25 (*p=*0.0001).

**Conclusion:**

Intravitreal Bevacizumab augmented with PRP is more effective in early regression of neovessels in high risk PDR patients.

## INTRODUCTION

Diabetes mellitus is a group of metabolic disorders which can lead to diabetic retinopathy in 80% of patients in the period of 20 years or more. Diabetic retinopathy has become one of the leading causes of loss of vision in under developing countries. Proliferative diabetic retinopathy (PDR) and diabetic macular edema (DME) are major causes of blindness and visual impairment in diabetic patients.^1^ Retinal ischemia and hypoxia results in release of vascular endothelial growth factors (VEGF) due to which there is an increase in the vascular permeability and proliferation of endothelial cells. The massive increase of VEGF in PDR results in the formation of neovessels at disc (NVD) and elsewhere NVE.^2^

In high risk PDR the main reasons for impairment of vision are vitreous hemorrhage, macular edema and tractional retinal detachment. The gold standard treatment for PDR is Panretinal photocoagulation (PRP) but it has many side effects like pain during procedure, macular edema or exacerbation in macular edema, peripheral visual loss, deterioration in visual fields, uveal effusion syndrome and defect in color vision.^1^,^2^, According to some recent studies the progression of macular edema is due to the accumulation of leukocytes at the non-coagulated posterior pole, post laser release of inflammatory mediators and angiogenic growth factors such as VEGF.^2^

Bevacizumab (avastin) is an anti-VEGF agent. It is a complete humanized monoclonal antibody that hamper various isoform of VEGF thereby regresses the Neovessels and stop episodes of rebleeding during the vitreous hemorrhage clearing process.^3^ As the duration of effect of anti VEGF is short, so they need to be repeated.^3^,^4^,^5^ Intravitreal Bevacizumab (IVB) is beneficial in case of neovascular glaucoma, persistent vitreous hemorrhage and before vitrectomy.^4^

This study aimed to compare the effectiveness of combination therapy of PRP augmented with anti VEGF and PRP alone in high risk PDR patients to assess the timing of regression of neovessels and improvement in visual acuity.

## METHODS

A randomized clinical control trial has been done at the department of Ophthalmology, ISRA University Hospital, Hyderabad between June 2018 to Dec 2018, after approval from ethical review committee [IUH/DEAN(CS)/205/17] and in accordance with tenets of declaration of Helsinki. Written Informed consent was taken from all patients entered the study.

### Inclusion criteria

All patients with Type-1 and Type-2 diabetes mellitus between 18 years to 65 years of age without any previous treatment.

### Exclusion Criteria

Patients with no prior history of intravitreal bevacizumab (IVB) or steroids, retinal laser therapy, vitrectomy, and patients with any media opacity like cataract, as it prevents the visualization of fundus.

### Treatment Protocol

We divided patients into two groups: Group-A in which only PRP was done, while in Group-B PRP plus intravitreal Bevacizumab was given. All patients undergone detailed ophthalmic examination which included visual acuity with log mar chart, Goldman’s applanation tonometry, dilated fundus examination with 90 D Volk lenses, fundus fluorescein angiography for neovessel regression time, optical coherence tomography (OCT- Topcon 3D) to calculate retinal thickness. Systemic investigation included fasting blood sugar, glycated hemoglobin Hb A1C, serum lipid profile and renal function test.

High-risk PDR according to ETDRS is defined as:

1. Neovascularization of the disc (NVD) more than 1/4 disc area;
2. vitreous or preretinal hemorrhage associated with any NVD; or
3. Neovascularization elsewhere (NVE) more than ½ disc area with vitreous or preretinal hemorrhage.

### Procedure

In Group A three sessions of PRP were done with o1 week interval, two thousand burns were applied at 1^st^ session with 200um spot size, duration of 20 msec pulse and from 400 to 500mW power to achieve greyish burns. Further PRP was done at one month and two months with supplementary 800-1000 burns. In Group B subjects first Intravitreal Bevacizumab injection (1.25mg/0.05ml) was given followed by PRP after 01^st^: week, likewise 02 more PRP episodes were done, second IVB injection was given at the end of 03^rd^ PRP session. The sessions of PRP and IVB injections were given by the second author. Post injection Topical antibiotics four times a day was given for 03 days.

### Primary Endpoint measures:

### 1. Timing of regression of neovessels: We have defined:

### I. Complete neovascular regression:

as total disappearance of former clinically active neovessels or total disappearance of neovessels from earlier fibrovascular membranes, leaving avascular membranes.

### II. Partial neovascular regression:

as reduction in the size or number of clinically active neovessels, and neovascular recurrence as formation of neovessels after complete regression^7^.

### 2. BCVA before and after treatment

-- CMT before and after treatment was secondary outcome measure in our study.

### Statistical Analysis

To study mean and standard deviation between two groups SPSS 21.0 version was used for the analysis of data. For analysis of pretreatment and post treatment BCVA as well as regression of NVE and NVDs and CMT within a group, paired sample t-test was used, while for calculation of induced change in BCVA, regression of NVE, NVD and CMT between the two groups (Group-A and Group-B) independent t- test (p < 0.05 significance level) was used.

## RESULTS

Seventy-six eyes of 52 patients were grouped into two groups having n=38 in each group. Age (year) in Group A was 50.7±6.9, and in Group B was 51.1±5.9. The male (%) and female (%) in Group-A was 58.25 and 41.75 respectively while 62.96 and 37.04 in Group-B respectively, other characteristics are shown in Table-I. Mean NVD and NVE status of both the groups at baseline, day 30, 3 months and 6 months are shown in Table-I.

**Table-I:** Baseline characteristics of patients (n=76).

Baseline characteristics	PRP group n=38 (Mean±SD)	PRP-Plus group n=38 (Mean±SD)
Age (years)	50.7±6.9	51.1±5.9
Male (%)	58.25	62.96
Female (%)	41.75	37.04
Duration of DM (years)	13±5	15±4.5
BCVA (logMAR)	0.35±0.60	0.34±0.6
Eyes with CSME (%)	43.13	47.14

**BCVA:** best corrected visual acuity, **CSME:** clinically significant macular oedema, **DM:** diabetic mellitus, **logMAR:** logarithm of minimum angle of resolution.

Mean BCVA worsened remarkably from mean 0.30±0.07 to mean 0.40±0.04 at 1 month, and mean 0.40±0.04 at 90^th^day in the PRP group. Though, mean BCVA improved from 0.30±0.05 to 0.1±0.03 at 1 month, and 0.1±0.02 at 3 months, in PRP-Plus group. Among the two groups there were highly remarkable changes at 1 month (*p*=0.00004) and at 3 months (*p*=0.00002) (Table-II).

**Table-II:** NVD, NVE, and BCVA status at presentation and follow up (n=76).

	PRP-Group n=38 (Mean±SD)	PRP Plus-IVB Group n=38 (Mean±SD)	P

NVD (DD%±SD)			
Baseline	40±5	40±7	-
4 weeks	50±7	10±5	0.00004
3 Months	40±6	11±3	0.00008
6 Months	40±4	12±2	0.00004
NVE (DD±SD)			
Baseline	2±0.75	2±0.50	-
4 weeks	2.25±0.75	01±0.5	0.0001
3 Months	2.00±0.50	0.75±0.25	0.0001
6 Months	2.10±0.60	0.70±0.35	0.0001
CVA (log MAR)			
Baseline	0.30±0.07	0.30±0.05	-
4 weeks	0.40±0.05	0.1±0.03	0.00004
3 Months	0.40±0.04	0.1±0.02	0.00002
6 Months	0.42±0.07	0.1±0.04	0.00002
CMT			
Baseline	250.5±10.3	253.91±15.2	0.393032
4 weeks	273.06±11.5	236.95±17.3	0.001116
3 Months	267±10.6	235.16±14.9	0.004973
6 Months	299±9.7	240.53±14.1	0.000011

**PRP:** panretinal photocoagulation, **PRP-plus:** panretinal photocoagulation plus intravitreal injection of 1.25 mg of bevacizumab, **NVD:** Neovessels on the disc, **NVE:** new vessels elsewhere, **DD:** Disc diameter, **CVA:** corrected visual acuity, **logMAR:** logarithm of minimum angle of resolution.

## DISCUSSION

The PRP is the main treatment for the PDR but it takes about three months for complete regression of the neovessels and recurrence of neovessels occur after 1^st^ session of laser in about one third of patients and at many times it’s not even effective. Extensive vitreous hemorrhage prevents the laser photocoagulation hence it increases the complication of high-risk PDR.^6^,^7^ According to Protocol S study of diabetic retinopathy clinical research network two years for PDR.^8^ We in our study use the intravitreal anti-VEGF as adjunctive treatment along with PRP to compare the effectiveness in regression of neovessels in divided groups where in one group only PRP was done and in another group both IVB along with PRP was done.

Simunovicetal concluded in their study the use of anti-VEGF prior to PRP showed better results in structural and functional outcome at three to four months and if given prior to Pars plana vitrectomy (PPV) reduces duration of surgery, fewer breaks, and less intra-operative bleeding.^9^ We in our study observed better visual outcome at one month and three months in the combined group.

Yang CS et al. and Dervenis N et al. and others proved in their study that PRP augmented with IVB achieved better visual improvement, early resorption of VH along with NVE and NVD in the treatment of high-risk PDR.^10^-^15^ We also in our study found early regression of NVD and NVE at one month, three months, and six months in combined group.

Some authors described that intravitreal anti-VEGF was non-inferior to PRP in regard to more cost effective, visual field loss, lowered vitrectomy rate, visual acuity and development of DME.^12^-^14^ Our study says that IVB augmented with PRP show better results than PRP alone.

Many studies in literature have proved that IVB administered prior to vitrectomy in the management of high risk PDR has better clinical outcome, in regard of NVE and NVD regression along with reduction in intraoperative bleeding, incidence of recurrent vitreous hemorrhage (VH), and better functional outcome like visual acuity.^16^-^19^ In our study no such parameters were considered and long-term studies should be conducted to prove the role of IVB prior to vitrectomy.

Alagozetal C demonstrated in their study that in VH secondary to PDR IVB reduces requirement for surgery and increases the number of patients in which PRP could have applied in early period but no impact on vision.^20^,^21^ Whereas in our study we found a better visual outcome with PRP plus group.

In our study we found improvement in BCVA, regression of retinal neovessels and macular thickness in both the groups (Table-II), but timing of neovessels regression was earlier in combined group as compared to PRP alone group.

Some studies published in literature of ophthalmology previously showed that when there was total neovascular regression after four weeks of injection bevacizumab, to induce a more durable effect against neovascular recurrence and to avoid the side effects of repeated intravitreous injections they applied standard PRP.^22^-^25^ But in our study all neovascular recurrences occurred after the 4-week visit in PRP only group while there was no recurrence in PRP plus group, so, we suggest earlier intravitreal administration of bevacizumab in eyes with complete neovascular regression following PRP. Earlier PRP may protect against some neovascular recurrence after bevacizumab.

### Limitations of study

It has small sample size. Short term and long-term complications of intravitreal bevacizumab like subconjunctival hemorrhage, endophthalmitis, retinal detachment, cataract and raised intraocular pressure were not considered. Further additional work is required in the management of proliferative diabetic retinopathy.

## CONCLUSION

According to the improvement in results of BCVA and timing of regression of neovessels status after combined intravitreal bevacizumab injection and PRP demonstrated that intravitreal bevacizumab is a beneficial adjunctive treatment for high-risk PDR. Though multiple reinjections are required to maintain a visual improvement, and regression of retinal neovessels in some patients.

### Authors Contribution:

**R** conceived, designed and did statistical analysis & editing of manuscript, is responsible for integrity of research.

**FFS** did data collection and manuscript writing.

**SMJ** did review and final approval of manuscript.
